# Deep Learning–Based Chronic Obstructive Pulmonary Disease Exacerbation Prediction Using Flow-Volume and Volume-Time Curve Imaging: Retrospective Cohort Study

**DOI:** 10.2196/69785

**Published:** 2025-05-15

**Authors:** Eun-Tae Jeon, Heemoon Park, Jung-Kyu Lee, Eun Young Heo, Chang Hoon Lee, Deog Kyeom Kim, Dong Hyun Kim, Hyun Woo Lee

**Affiliations:** 1 Department of Neurology Korea University Ansan Hospital Korea University College of Medicine Ansan Republic of Korea; 2 Department of Internal Medicine Division of Respiratory and Critical Care Seoul National University College of Medicine, Seoul Metropolitan Government-Seoul National University Boramae Medical Center Seoul Republic of Korea; 3 Department of Internal Medicine Division of Pulmonary and Critical Care Medicine Seoul National University Hospital Seoul Republic of Korea; 4 Department of Radiology Seoul Metropolitan Government-Seoul National University Boramae Medical Center Seoul National University College of Medicine Seoul Republic of Korea

**Keywords:** pulmonary disease, chronic obstructive, artificial intelligence, respiratory function tests, spirometry, symptom flare up

## Abstract

**Background:**

Chronic obstructive pulmonary disease (COPD) is a common and progressive respiratory condition characterized by persistent airflow limitation and symptoms such as dyspnea, cough, and sputum production. Acute exacerbations (AE) of COPD (AE-COPD) are key determinants of disease progression; yet, existing predictive models relying mainly on spirometric measurements, such as forced expiratory volume in 1 second, reflect only a fraction of the physiological information embedded in respiratory function tests. Recent advances in artificial intelligence (AI) have enabled more sophisticated analyses of full spirometric curves, including flow-volume loops and volume-time curves, facilitating the identification of complex patterns associated with increased exacerbation risk.

**Objective:**

This study aimed to determine whether a predictive model that integrates clinical data and spirometry images with the use of AI improves accuracy in predicting moderate-to-severe and severe AE-COPD events compared to a clinical-only model.

**Methods:**

A retrospective cohort study was conducted using COPD registry data from 2 teaching hospitals from January 2004 to December 2020. The study included a total of 10,492 COPD cases, divided into a development cohort (6870 cases) and an external validation cohort (3622 cases). The AI-enhanced model (AI-PFT-Clin) used a combination of clinical variables (eg, history of AE-COPD, dyspnea, and inhaled treatments) and spirometry image data (flow-volume loop and volume-time curves). In contrast, the Clin model used only clinical variables. The primary outcomes were moderate-to-severe and severe AE-COPD events within a year of spirometry.

**Results:**

In the external validation cohort, the AI-PFT-Clin model outperformed the Clin model, showing an area under the receiver operating characteristic curve of 0.755 versus 0.730 (*P*<.05) for moderate-to-severe AE-COPD and 0.713 versus 0.675 (*P*<.05) for severe AE-COPD. The AI-PFT-Clin model demonstrated reliable predictive capability across subgroups, including younger patients and those without previous exacerbations. Higher AI-PFT-Clin scores correlated with elevated AE-COPD risk (adjusted hazard ratio for Q4 vs Q1: 4.21, *P*<.001), with sustained predictive stability over a 10-year follow-up period.

**Conclusions:**

The AI-PFT-Clin model, by integrating clinical data with spirometry images, offers enhanced predictive accuracy for AE-COPD events compared to a clinical-only approach. This AI-based framework facilitates the early identification of high-risk individuals through the detection of physiological abnormalities not captured by conventional metrics. The model’s robust performance and long-term predictive stability suggest its potential utility in proactive COPD management and personalized intervention planning. These findings highlight the promise of incorporating advanced AI techniques into routine COPD management, particularly in populations traditionally seen as lower risk, supporting improved management of COPD through tailored patient care.

## Introduction

Chronic obstructive pulmonary disease (COPD) is a common and progressive respiratory disorder characterized by persistent airflow limitation, associated risk factors, and a range of symptoms, including dyspnea, sputum production, and chronic cough. Its development is influenced by multiple well-recognized risk factors, including smoking, occupational exposures, environmental pollutants, and early-life lung development abnormalities [1]. As a leading cause of morbidity and mortality worldwide, COPD presents significant challenges, particularly in the management of acute exacerbation (AE) and acute exacerbation of COPD (AE-COPD). AE-COPD is a critical event that significantly influences disease progression, quality of life, health care usage, and mortality [2]. Current COPD treatment guidelines emphasize optimized management to prevent AE-COPD in high-risk patients. Although various risk factors for AE-COPD have been identified, 2 key features—symptoms and a previous history of exacerbations—are most commonly used to stratify patients for treatment decisions [3]. Tailored based on these features, inhaled treatments for stable COPD have significantly reduced the incidence of AE-COPD [4].

Lung function is a crucial marker of COPD progression and prognosis [5]. Spirometry provides essential information on lung function, such as forced expiratory volume in 1 second (FEV_1_), which has been used to assess the degree of airflow limitation or disease stages. Previous studies have demonstrated an independent association between FEV_1_ and the risk of AE-COPD in both young and old patients with COPD [6,7]. FEV_1_ is a key predictor of AE-COPD in the majority of existing prediction models [8]. However, its association with exacerbations is relatively weak, limiting its role as a predictive factor [9]. Its contribution to improving predictive performance for future AE-COPD has been minimal [10,11], likely because FEV_1_ captures only a fraction of the information available from spirometry results.

Recent advances in artificial intelligence (AI) enhance predictive accuracy and early identification of high-risk patients. AI can analyze spirometric data's complex features, potentially surpassing traditional methods in classifying COPD subtypes [12]. By examining spirometry curves, AI models may uncover insights into AE-COPD prediction that conventional measures miss.

Therefore, this study aims to develop and validate an AI model that integrates clinical data and comprehensive spirometric information, including flow-volume loop and volume-time curve analyses, to predict the occurrence of AE-COPD within 1 year.

## Methods

Our study followed the Strengthening the Reporting of Observational Studies in Epidemiology (STROBE) guidelines [13].

### Study Design and Participants

This retrospective cohort study analyzed the individuals who were regularly followed up for COPD management at 2 teaching hospitals (Boramae Medical Center [BRMH] and Seoul National University Hospital [SNUH]) from January 2004 to December 2020. We included the patients with COPD who (1) had chronic respiratory symptoms including dyspnea, sputum, and cough; (2) performed postbronchodilator spirometry with an FEV_1_/forced vital capacity (FVC) ratio of less than 0.7; (3) visited the outpatient clinic at least annually; and (4) had medical records regarding AE events in the past and during follow-up. Exclusion criteria included the presence of acute pulmonary conditions at baseline, such as pneumonia, acute pulmonary edema, or pulmonary embolism. Patients with interstitial lung disease, including idiopathic pulmonary fibrosis and connective tissue disease-associated interstitial lung disease, were excluded. In addition, patients with active malignancies (eg, lung cancer, metastatic solid tumors, and hematologic malignancies) and those with severe nonpulmonary comorbidities (eg, end-stage renal disease, end-stage heart failure, decompensated liver cirrhosis, and neuromuscular disorders affecting respiratory function) were excluded.

### Data Preparation

**Figure 1:**
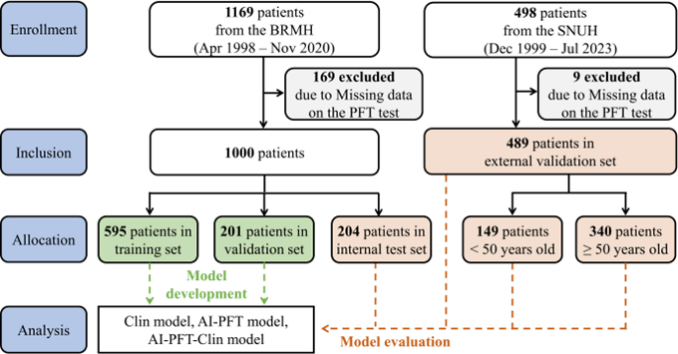
Flow diagram of patient selection. AI: artificial intelligence; BRMH: Boramae Medical Center; PFT: pulmonary function test; SNUH: Seoul National University Hospital.

Each pulmonary function test (PFT) was treated as an independent observation rather than selecting a single record per patient. To maximize data usage while preserving the integrity of model evaluation, all PFT records from the same patient were assigned to a single dataset—either training, validation, or test—rather than being distributed across multiple datasets. This patient-level partitioning strategy was implemented to prevent data leakage, minimize overfitting, and ensure that the model’s predictive performance was evaluated on independent patient cohorts. The flow-volume loop and volume-time curve were extracted from the graphic spirometry data and used for model development. Patients from BRMH were divided into the training set (595/1000, 60%), internal validation set (201/1000, 20%), and internal test set (204/1000, 20%) with stratification based on outcomes and subgroup variables. Patients from SNUH were used to form the external validation set, with groups categorized based on age (Figure 1). Details regarding the variables, spirometric measurement, data extraction, and data preprocessing processes are provided in Multimedia Appendix 1 [14,15].

### Outcomes

The primary outcomes were moderate-to-severe and severe AE-COPD events occurring within 1 year after the spirometry test. A moderate AE-COPD was defined as a worsening of COPD symptoms necessitating the use of antibiotics or systemic corticosteroids. A severe AE-COPD was defined by the requirement for hospitalization or emergency department visits due to symptom flare-ups. These outcomes were analyzed for the total patient cohort and stratified by age (≥50 or <50 years). The 50-year cutoff was selected based on the global initiative for chronic obstructive lung disease expert consensus, which defines young patients with COPD as those under 50 years [16].

Subgroup analyses were conducted to assess the AI model’s performance based on history of moderate-to-severe exacerbations, presence of dyspnea, use of inhaled treatments, and type of inhaled treatment regimen. As a sensitivity analysis, the time to moderate-to-severe exacerbation event was assessed according to the quartile of AI-PFT-Clin scores, and changes in the AI-PFT-Clin score based on the presence or absence of exacerbations were evaluated over time.

### Model Development

We developed a 1D transformer-based model [14], named the “AI-PFT model,” which processes volume-time curves and flow-volume loops to predict the outcomes. The model’s output score was referred to as the “AI-PFT score.” To effectively use spirometry data, volume-time curves, and flow-volume loops were each processed independently before being integrated into the final predictive framework. The flow-volume loops were segmented into patches to capture both global respiratory patterns and localized variations in airflow, while the volume-time curves were structured to preserve temporal dependencies in expiratory flow dynamics. Each representation was then encoded using a Transformer architecture using multihead self-attention mechanisms, which enabled the model to learn both short-range and long-range dependencies within the respiratory signal. The encoded feature representations from flow-volume loops and volume-time curves were subsequently combined using an additive fusion approach, ensuring that both inputs contributed to the final prediction without loss of relevant physiological information. The final prediction layer consisted of a multilayer perceptron with 2 output heads, each responsible for generating probability scores for moderate-to-severe AE-COPD risk and severe AE-COPD risk.

Details on the model's architecture and hyperparameters of the AI model are presented in Multimedia Appendices 1 and Figure S1 in Multimedia Appendix 2.

The “Clin model” and “AI-PFT-Clin model” were developed through a multivariable logistic regression approach and their output was referred to as the “Clin score” and “AI-PFT-Clin score,” respectively. The Clin model incorporates only clinical variables that are both associated with exacerbation risk and serve as key factors in guiding current COPD management, including a history of moderate or severe AE-COPD, dyspnea, and the use of long-acting beta-agonists (LABA), and long-acting muscarinic antagonists (LAMA) and inhaled corticosteroids (ICS). The AI-PFT-Clin model integrated the clinical variables and the AI-PFT score.

### Statistical Analysis

Descriptive statistics are presented as numbers (percentages), means (SD), or medians (IQR). Normality was assessed using the Shapiro-Wilk test and homoscedasticity was checked with the Levene test. Group comparisons were conducted using the chi-square test, Student *t* test, or Mann-Whitney *U* test.

The area under the receiver operating characteristic curve (AUROC) was the primary evaluation metric, calculated using the DeLong method [17]. For each model, the cutoff value was determined using the Youden index from the internal validation set, and sensitivity, specificity, positive predictive value, and negative predictive value were evaluated. The AI-PFT-Clin score was used to stratify each evaluation set into 4 quartiles. Cox proportional hazards regression analysis was conducted to assess the predictive power of the AI-PFT-Clin model, both in univariable models and in multivariable models adjusted for age, sex, BMI, smoking status, emphysema, Charlson comorbidity index, white blood cell count, neutrophil count, lymphocyte count, eosinophil count, blood urea nitrogen, creatinine, protein, and albumin. Kaplan-Meier survival curves were plotted for the quartile strata, and pairwise comparisons using the log-rank test were performed to evaluate the model’s ability to stratify AE risk.

The significance level was set at *P*<.05. All analyses, including model development and statistical analysis, were executed in Python (version 3.10.13; Python Software Foundation), Tensorflow (version 2.15.0; Google Brain Team), and Keras (version 3.1.1; Oneiros).

### Ethical Considerations

In accordance with the Declaration of Helsinki, our study was conducted with adherence to ethical standards. This study received approval (institutional review board number 20-2023-11) from the institutional review board of Seoul Metropolitan Government-Seoul National University BRMH, which exempted the need for written informed consent.

## Results

### Overview

A total of 1169 patients were initially enrolled in BRMH between April 1998 and November 2020, with 169 excluded due to missing PFT data. Similarly, 598 patients were enrolled from SNUH between December 1999 and July 2023, with 9 excluded for the same reason. The median follow-up duration was 79 (IQR 43-127) months for the BRMH cohort and 138 months (IQR 94-194) months for the SNUH cohort.

### Baseline Characteristics of the Participants

The baseline characteristics of the 1489 patients (1000 from BRMH and 489 from SNUH) showed that BRMH patients were older, predominantly male, and had higher rates of smoking, cough, dyspnea, emphysema, and moderate-to-severe AE-COPD, along with a higher Charlson comorbidity index (Table 1). In contrast, SNUH patients had higher blood eosinophil counts, lower post-bronchodilator FEF_25%-75%_, and more frequent use of ICS. The dataset comprised 6870 PFT cases from the patients at BRMH and 3622 cases from patients at SNUH. Within the SNUH group, 794 PFT cases were derived from 149 patients with COPD under the age of 50, and 2828 cases from 340 patients with COPD aged 50 years and older. Detailed characteristics of the internal test set and the subgroups of the external validation set, divided by age under and over 50 years, are provided in Table S1 in Multimedia Appendix 2.

**Table 1:** Baseline characteristics of the study population.

Variable		All (n=1489)	BRMH^a^ (n=1000)	SNUH^b^ (n=489)	*P* value
**Demographics**					
	Age (years), median (IQR)	62 (49-69)	64 (55-71)	57 (45-66)	<.001
	Male, n (%)	1200 (80.6)	864 (86.4)	336 (68.7)	<.001
	BMI, kg/m^2^, median (IQR)	22.4 (20.7-24.4)	22.5 (20.1-24.8)	22.4 (21.8-23.3)	.63
**Smoking**					
	Never smoker, n (%)	334 (22.4)	198 (19.8)	136 (27.8)	<.001
	Ex-smoker, n (%)	747 (50.2)	429 (42.9)	318 (65.0)	<.001
	Current smoker, n (%)	408 (27.4)	373 (37.3)	35 (7.2)	<.001
	Pack-year in ever-smokers, median (IQR)	30.0 (10.0-40.0)	30.0 (10.0-45.0)	29.1 (12.8-35.8)	<.001
**Respiratory symptoms, n (%)**					
	Cough	463 (31.1)	407 (40.7)	56 (11.5)	<.001
	Sputum	661 (44.4)	564 (56.4)	97 (19.8)	<.001
	Dyspnea	1248 (83.8)	853 (85.3)	395 (80.8)	.03
History of moderate-to-severe AE-COPD^c^, n (%)		726 (48.8)	426 (42.6)	300 (61.3)	<.001
Charlson comorbidity index, median (IQR)		2 (1-2)	2 (1-3)	1 (1-2)	<.001
**Laboratory findings, median (IQR)**					
	Blood WBC^d^, 10^3^/μL	7664 (6280-8576)	7494 (6086-9080)	7684 (7192-7788)	.56
	Blood neutrophil, predicted %	60.5 (54.9-65.9)	60.4 (53.8-69.4)	60.6 (57.9-61.4)	.06
	Blood neutrophil count, 10^3^/μL	4780 (3532-5376)	4516 (3328-5884)	4812 (4038-4860)	.77
	Blood lymph, predicted %	28.9 (23.7-33.9)	29.0 (20.8-35.1)	28.9 (28.2-30.2)	.33
	Blood lymph count, 10^3^/μL	2094 (1646-2432)	2068 (1539-2557)	2098 (2000-2196)	.42
	Blood eosinophil, predicted %	2.82 (1.40-3.70)	2.40 (1.10-4.00)	2.99 (2.55-3.34)	<.001
	Blood eosinophil count, 10^3^/μL	199 (103-268)	176 (88-288)	213 (176-244)	<.001
	BUN^e^, mg/dL	14.3 (12.0-16.0)	14.0 (12.0-16.2)	14.5 (12.0-15.5)	.48
	Creatinine, mg/dL	0.91 (0.80-0.97)	0.90 (0.80-1.00)	0.92 (0.82-0.94)	.02
	Total bilirubin, mg/dL	0.74 (0.60-0.80)	0.72 (0.60-0.80)	0.75 (0.70-0.75)	.16
	Blood protein, g/dL	7.00 (6.70-7.30)	7.00 (6.60-7.30)	7.06 (6.97-7.30)	<.001
	Blood albumin, g/dL	4.10 (3.90-4.30)	4.10 (3.90-4.30)	4.09 (4.02-4.30)	<.001
**Radiologic findings, n (%)**					
	Emphysema	656 (44.1)	583 (58.3)	73 (14.9)	<.001
	TDL^f^	100 (6.7)	80 (8.0)	20 (4.1)	.007
**Pulmonary function tests**					
	Post-BDR^g^ FEV_1_^h^, L	1.71 (1.34-2.14)	1.74 (1.27-2.21)	1.69 (1.45-1.99)	.67
	Post-BDR FEV_1_, predicted %	66.9 (56.0-79.0)	69.0 (53.0-82.0)	66.2 (58.0-68.9)	<.001
	Post-BDR FVC^i^, L	3.20 (2.66-3.74)	3.21 (2.54-3.81)	3.20 (2.86-3.57)	.66
	Post-BDR FVC, predicted %	85.5 (73.0-95.0)	88.0 (75.0-99.2)	83.4 (68.0-87.4)	<.001
	Post-BDR FEV_1_/FVC, predicted %	56.0 (49.0-64.0)	57.0 (47.0-66.0)	55.6 (53.3-59.1)	.09
	Post-BDR FEF_25-75%_, L/sec	0.81 (0.54-1.12)	0.76 (0.48-1.17)	0.86 (0.73-1.04)	<.001
	Post-BDR FEF_25-75%_, predicted %	31.0 (21.0-40.0)	30.0 (19.0-43.0)	31.2 (28.5-34.6)	.33
	DL_CO_^j^, predicted %	83.0 (71.0-94.6)	83.0 (68.0-97.0)	82.3 (74.8-90.0)	.52
	DL_CO_/VA^k^, predicted %	90.0 (76.0-106.0)	87.0 (70.0-104.0)	96.6 (82.4-109.5)	<.001
**Inhaled treatments, n (%)**					
	LABA^l^	1061 (71.3)	704 (70.4)	357 (73.0)	.33
	LAMA^m^	1157 (77.7)	759 (75.9)	398 (81.4)	.02
	ICS^n^	923 (62.0)	555 (55.5)	368 (75.3)	<.001

^a^BRMH: Boramae Medical Center.

^b^SNUH: Seoul National University Hospital.

^c^AE-COPD: acute exacerbations of chronic obstructive pulmonary disease.

^d^WBC: white blood cell.

^e^BUN: blood urea nitrogen.

^f^TDL: tuberculosis destroyed lung.

^g^BDR: bronchodilator response.

^h^FEV_1_: forced expiratory volume in 1 second.

^i^FVC: forced vital capacity.

^j^DL_CO_: diffusing capacity of the lung for CO.

^k^VA: alveolar volume.

^l^LABA: long-acting beta-agonist.

^m^LAMA: long-acting muscarinic antagonist.

^n^ICS: inhaled corticosteroid.

### Overall Predictive Performance of the Models

**Figure 2:**
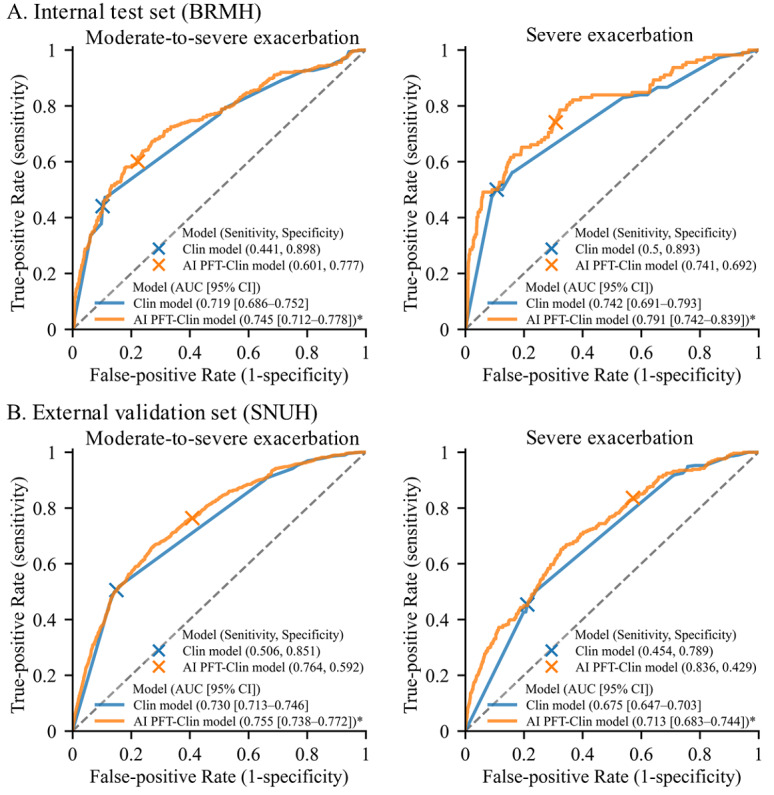
Receiver operating characteristics curves for prediction of moderate-to-severe and severe exacerbations. BRMH: Boramae Medical Center, SNUH: Seoul National University Hospital; an asterisk (*) indicates significantly higher AUROC than the Clin model (*P*<.05).

Within 1 year post PFT, the BRMH cohort reported (1340/6870, 19.5%) moderate-to-severe and (544/6870, 7.9%) severe exacerbations, while the SNUH cohort reported (1038/3622 28.7%) and (293/3622, 8.1%), respectively. When comparing the performance of the Clin model and the AI-PFT model, the AI-PFT model demonstrated either a lower or comparable AUROC for predicting moderate-to-severe and severe AE-COPD, while exhibiting lower specificity and higher sensitivity across both outcomes (Table S2 in Multimedia Appendix 2). However, the results showed that the AI-PFT-Clin model significantly improved the predictive performance for both moderate-to-severe and severe AE-COPD compared to the Clin model (Figure 2). In the external validation set, the Clin model achieved AUROC values of 0.730 for moderate-to-severe AE-COPD and 0.675 for severe AE-COPD, while the AI-PFT-Clin model significantly increased these to 0.755 and 0.713, respectively (both *P*<.05). In addition, patients who experienced exacerbations showed significantly higher AI-PFT-Clin scores (Figure S2 in Multimedia Appendix 2).

The AI-PFT-Clin model showed a higher AUROC for predicting moderate-to-severe AE-COPD regardless of previous history of exacerbation, whereas its performance was not significantly different in groups without dyspnea or the use of inhaled treatments like ICS, LABA, or LABA/LAMA (Figure S3 in Multimedia Appendix 2). Similarly, for severe exacerbations, the AI-PFT-Clin model showed better predictive accuracy compared to the Clin model regardless of history of exacerbation or inhaled treatments, but this improvement was not observed in groups without dyspnea or ICS use (Figure S4 in Multimedia Appendix 2).

### Predictive Performance of the Models According to Age

**Figure 3:**
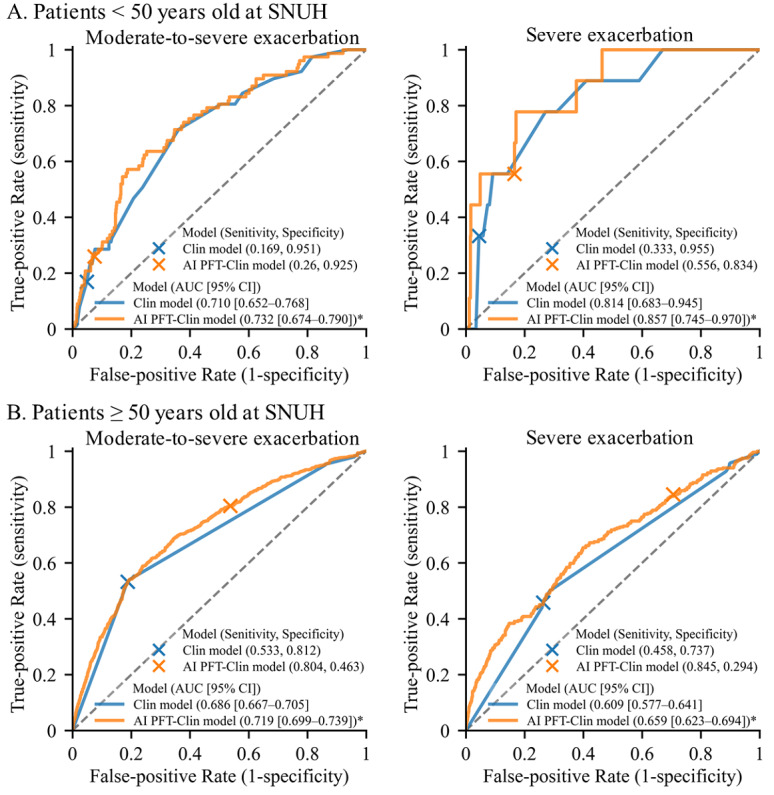
Receiver operating characteristics curves for prediction of moderate-to-severe and severe exacerbations according to age. SNUH: Seoul National University Hospital; an asterisk (*) indicates significantly higher AUROC than the Clin model (*P*<.05).

The predictive performance of the 3 different models across age-based subgroups is described (Table S3 in Multimedia Appendix 2). Across both age subgroups, the AI-PFT-Clin model consistently improved predictive performance compared to the Clin model (Figure 3). In patients younger than 50 years old, the Clin model achieved AUROC values of 0.710 for moderate-to-severe AE-COPD and 0.814 for severe AE-COPD, while the AI-PFT-Clin model significantly improved these values to 0.732 and 0.857, respectively (both *P*<.05). In patients aged 50 years and older, the Clin model yielded AUROC values of 0.686 for moderate-to-severe AE-COPD and 0.609 for severe AE-COPD, while the AI-PFT-Clin model increased these to 0.719 and 0.659, respectively (both *P*<.05).

In both patients younger than 50 and those aged 50 years and older, the AI-PFT-Clin model demonstrated higher AUROC values for predicting moderate-to-severe AE-COPD, with significantly better performance in subgroups with dyspnea or receiving inhaled treatments like LABA, LAMA, or LABA/LAMA (Table S4 in Multimedia Appendix 2). However, for severe exacerbations, the AI-PFT-Clin model’s performances in different subgroups varied by age (Figure S5 in Multimedia Appendix 2). In patients younger than 50 years old, the model showed higher AUROC values for predicting severe exacerbations only in subgroups with a history of AEs, with dyspnea, but without treatments such as LAMA. In contrast, for patients aged 50 years and older, the AI-PFT-Clin model showed better predictive accuracy for severe exacerbations in subgroups with dyspnea and those on inhaled treatments.

### Time to AE Event and Cox Proportional Hazards Regression Results

**Figure 4:**
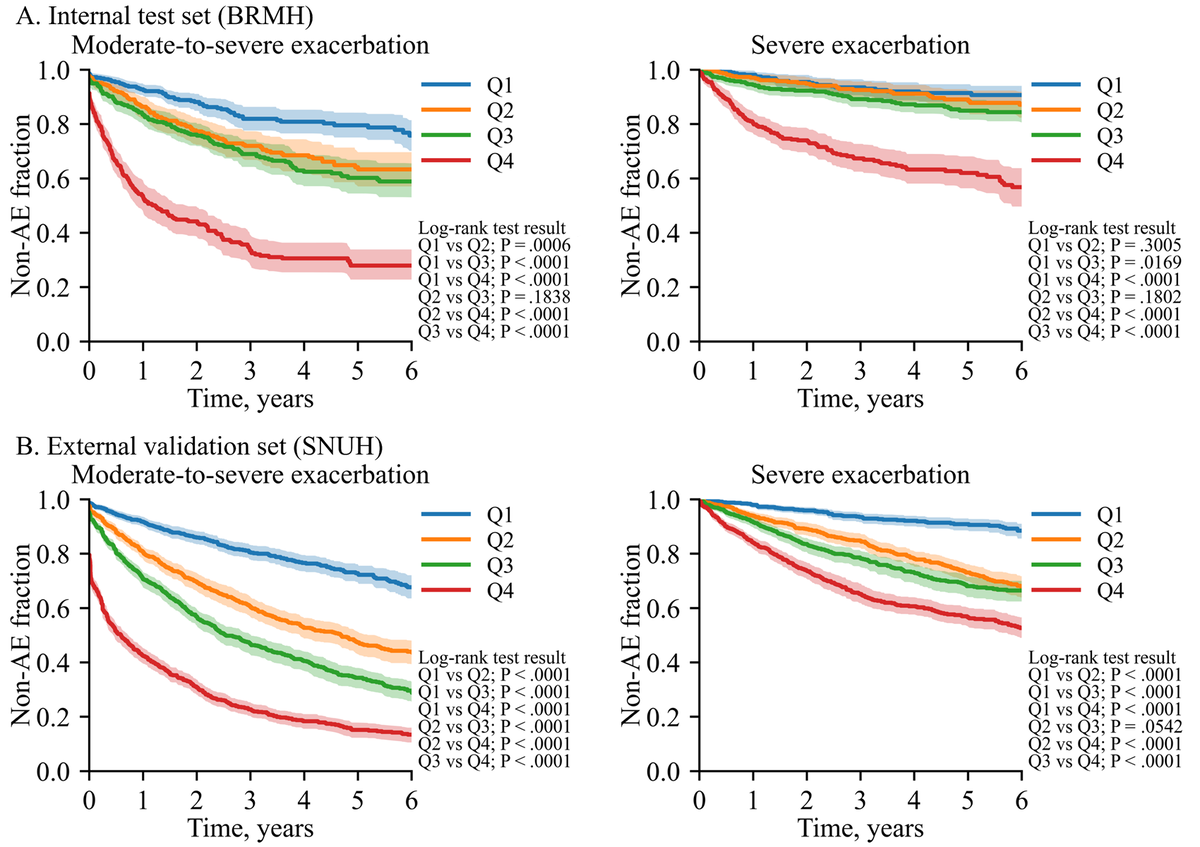
Time to exacerbation event according to AI-PFT-Clin score. BRMH: Boramae Medical Center, SNUH: Seoul National University Hospital.

The Kaplan-Meier survival curves demonstrate a clear trend of increasing risk of AEs across AI-PFT-Clin score quartiles, with higher quartiles experiencing faster onset of moderate-to-severe and severe AE-COPD (Figure 4). Notably, patients in the highest quartile (Q4) group exhibited a significantly greater risk of earlier AEs compared to those in other groups (all comparisons, *P*<.001). This pattern was consistent across both age groups, younger than and older than 50 years, with significant differences observed between the Q4 group and the other groups for both moderate-to-severe and severe AE-COPD (all comparisons, *P*<.05, Figure S6 in Multimedia Appendix 2).

In figure 4, AI-PFT-Clin score was used to obtain the 4 strata. According to the prediction score, Q1, Q2, Q3, and Q4 denote the first, second, third, and fourth quartiles. Solid lines and shades represent mean curves and 95% CI areas, respectively. Pair-wise comparisons between the curves were performed and *P* values are presented in the legend.

The analysis of adjusted hazard ratios (aHRs) demonstrated a consistent increase in AE risk with higher AI-PFT-Clin quartiles for both moderate-to-severe and severe AE-COPD (Table 2). For moderate-to-severe AE-COPD, the aHRs for the 2nd, 3rd, and 4th quartiles were 2.085, 3.544, and 4.217, respectively (*P*<.001 for all quartiles). Similarly, for severe AE-COPD, the aHRs were 2.289, 3.102, and 3.516 for the 2nd, 3rd, and 4th quartiles, respectively (*P*<.001 for all quartiles). While similar results were observed in patients aged 50 years and older, the association between the AI-PFT-Clin score and AEs was especially pronounced in the 3rd and 4th quartiles for patients younger than 50 years.

**Table 2:** Hazard ratio (HR) for acute exacerbation according to the quartile of AI-PFT-Clin score.

				Univariable					Multivariable^a^		
				HR (95% CI)		*P* value		HR (95% CI)			*P* value
**Moderate-to-severe AE^b^**											
	**All patients (reference: first quartile of AI-PFT-Clin score)**										
		Second quartile of AI-PFT-Clin score	2.354 (1.992-2.782)		<.001		2.085 (1.526-2.849)			<.001	
		Third quartile of AI-PFT-Clin score	3.476 (2.959-4.084)		<.001		3.544 (2.542-4.943)			<.001	
		Fourth quartile of AI-PFT-Clin score	7.134 (6.100-8.343)		<.001		4.217 (3.076-5.782)			<.001	
	**Patients <50 years old (reference:** **first** **quartile of AI-PFT-Clin score)**										
		Second quartile of AI-PFT-Clin score	2.474 (1.765-3.469)		<.001		1.061 (0.463-2.431)			.889	
		Third quartile of AI-PFT-Clin score	4.778 (3.572-6.390)		<.001		2.722 (1.054-7.027)			.039	
		Fourth quartile of AI-PFT-Clin score	10.245 (7.827-13.409)		<.001		3.958 (1.938-8.085)			<.001	
	**Patients ≥50 years old (reference:** **first** **quartile of AI-PFT-Clin score)**										
		Second quartile of AI-PFT-Clin score	1.070 (0.861-1.330)		.54		1.605 (1.087-2.370)			.02	
		Third quartile of AI-PFT-Clin score	1.517 (1.226-1.878)		<.001		2.151 (1.433-3.227)			<.001	
		Fourth quartile of AI-PFT-Clin score	3.073 (2.492-3.789)		<.001		2.232 (1.482-3.361)			<.001	
**Severe AE**											
	**All patients (reference: first quartile of AI-PFT-Clin score)**										
		Second quartile of AI-PFT-Clin score	2.919 (2.254-3.780)		<.001		2.289 (1.557-3.366)			<.001	
		Third quartile of AI-PFT-Clin score	3.471 (2.688-4.482)		<.001		3.102 (1.973-4.876)			<.001	
		Fourth quartile of AI-PFT-Clin score	5.593 (4.373-7.154)		<.001		3.516 (2.356-5.250)			<.001	
	**Patients <50 years old (reference: first quartile of AI-PFT-Clin score)**										
		Second quartile of AI-PFT-Clin score	3.303 (1.633-6.684)		<.001		3.700 (1.498-9.143)			.003	
		Third quartile of AI-PFT-Clin score	4.926 (2.569-9.445)		<.001		5.505 (1.943-15.596)			.001	
		Fourth quartile of AI-PFT-Clin score	10.203 (5.670-18.363)		<.001		8.585 (4.044-18.225)			<.001	
	**Patients ≥50 years old (reference: first quartile of AI-PFT-Clin score)**										
		Second quartile of AI-PFT-Clin score	1.193 (0.897-1.585)		.22		1.898 (1.166-3.092)			.01	
		Third quartile of AI-PFT-Clin score	1.403 (1.057-1.861)		.02		2.144 (1.237-3.716)			.007	
		Fourth quartile of AI-PFT-Clin score	2.184 (1.661-2.872)		<.001		2.389 (1.408-4.055)			.001	

^a^Multivariable Cox regression analysis included the AI-PFT-Clin score as well as age, sex, body mass index, smoking status, emphysema, Charlson comorbidity index, white blood cell count, neutrophil count, lymphocyte count, eosinophil count, blood urea nitrogen, creatinine, protein, and albumin as covariables.

^b^AE: acute exacerbation.

### AI-PFT-Clin Scores Over a 10-Year Follow-Up Period

**Figure 5:**
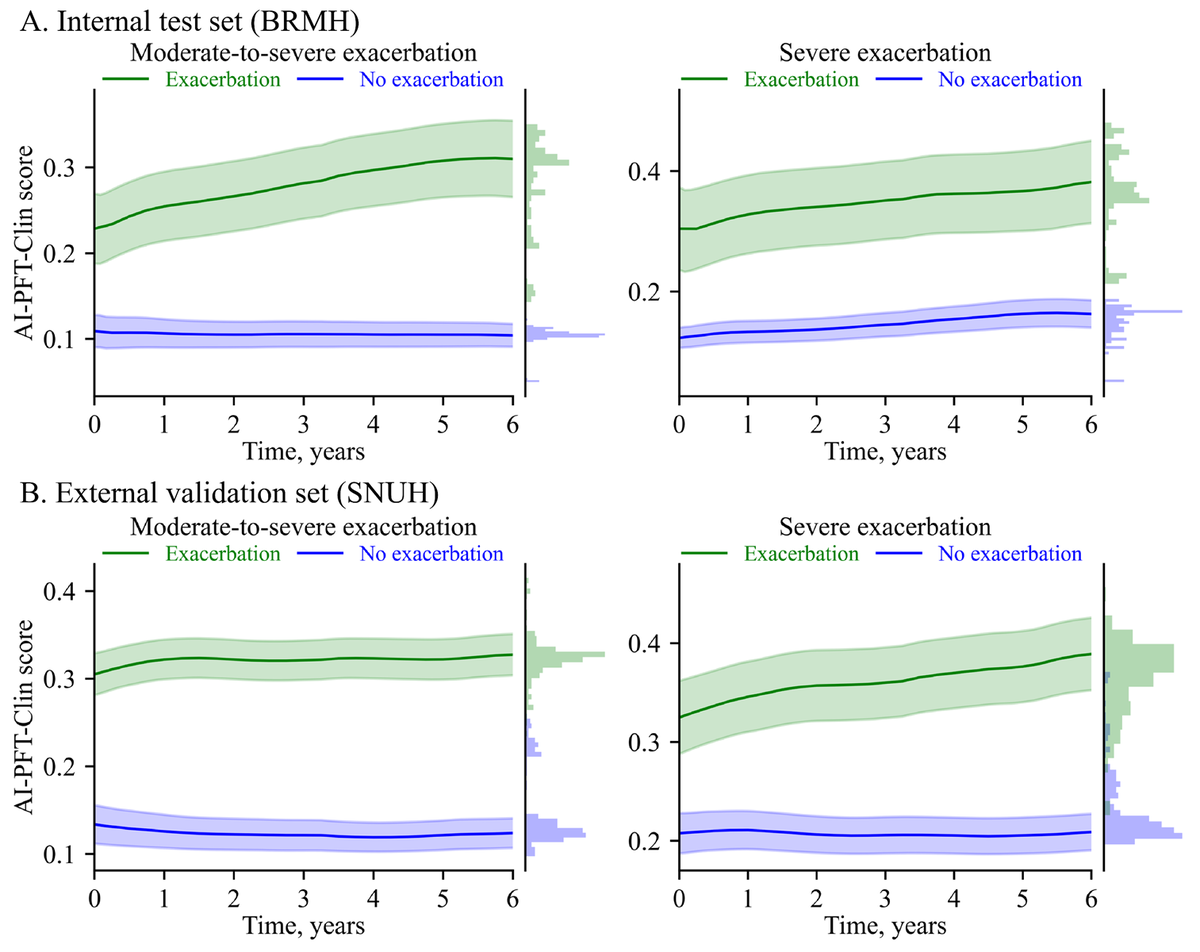
Predicted probability trajectories of exacerbation over 6 years.

The predicted probabilities for moderate-to-severe and severe AE-COPD over a 10-year follow-up period, based on the AI-PFT-Clin score, are described in Figure 5. Patients who experienced AEs consistently exhibited higher predicted probabilities throughout the 10-year period, whereas those without AEs maintained lower predicted probabilities for both moderate-to-severe and severe AE-COPD. This trend was consistent across both age groups, younger than and older than 50 years (Figure S7 in Multimedia Appendix 2).

In Figure 5, the predicted probabilities were obtained from the AI-PFT-Clin model. Solid lines and shades represent mean curves and 95% CI areas, respectively. Histograms on the right axis indicate distributions of the predicted probability.

## Discussion

### Principal Findings

This study developed and validated an AI model that integrates comprehensive spirometric data and clinical information to predict the risk of moderate-to-severe and severe AE-COPD within 1 year. To the best of our knowledge, this is the first study to integrate clinical data with quantitative spirometric imaging features to predict AE-COPD. The AI-PFT-Clin model significantly outperformed the Clin model in predicting both moderate-to-severe and severe AE-COPD in the external validation cohort. Importantly, the AI-PFT-Clin model demonstrated consistently superior predictive performance regardless of previous AE-COPD history or the initiation of inhaled treatment, highlighting its potential for identifying high-risk patients with COPD who have not yet experienced exacerbations or begun treatment. In addition, the model showed enhanced performance in patients younger than 50 years of age, indicating its promise in predicting AE-COPD in young COPD populations. Given that younger patients with COPD often exhibit distinct clinical characteristics, such as a higher prevalence of asthma history and previous pulmonary infections [6], we performed a subgroup analysis stratified by the 50-year cutoff to assess potential differences in model performance across epidemiologically distinct COPD phenotypes. AI-PFT-Clin score consistently distinguished between high- and low-risk patients over 6 years, rather than being limited to a single baseline prediction. The findings indicate that patients who experienced AE-COPD had higher AI-PFT-Clin scores in each respective year, reinforcing the longitudinal robustness of the model’s predictive ability.

Previous studies on AE-COPD prediction have primarily relied on traditional statistical models such as logistic regression or the Cox proportional hazards model [8]. The CODEX index and BODE index, which incorporate clinical and spirometric variables, are representative tools [18,19]. However, their predictive accuracy is limited when applied to heterogeneous COPD populations. The Acute COPD Exacerbation Prediction Tool, which integrates multiple clinical factors such as exacerbation history, lung function, and medication use, has shown promising predictive power with an AUC of 0.81 for predicting at least 2 exacerbations per year in external validation [11]. Nevertheless, its performance declines in patients without a history of exacerbations. In addition, shallow learning models like random forests and support vector machines have been used to predict AE-COPD using clinical data, including spirometry. While accuracy has improved, challenges like overfitting, missing data handling, and limited validation hinder their clinical use [20].

Recently, deep learning models, particularly convolutional neural networks (CNNs) and recurrent neural networks, have shown potential in leveraging complex data structures such as imaging and time-series data. Several studies have used conventional spirometric parameters such as FEV_1_, FVC, and FEF_25-75%_, processed through multilayer perceptron or used partial raw data points from the expiratory flow-volume curve using fully connected 1D CNNs [12,21]. However, these approaches were limited by their reliance on discrete segments of the respiratory curve, restricting their ability to capture continuous and complex dependencies between data points. In addition, other studies have used CNNs to analyze raw spirometric graphs or down-sampled binary images, but these methods risk introducing noise due to variability in raw graphical data, and aggressive down-sampling can lead to significant information loss [22-24]. In addition, they encountered issues related to overfitting and the need for large, high-quality datasets [20].

Our study presents several key novelties in comparison to previous approaches. Instead of using raw spirometry images that may contain unnecessary artifacts, we used a systematic extraction and digitization process to convert flow-volume loops and volume-time curves into structured numerical data. This transformation ensured that only the most relevant physiological information was used, reducing potential noise and improving model robustness. Furthermore, our model architecture leveraged a Transformer-based framework, which is particularly well-suited for processing time series and spatially structured data. Unlike traditional machine learning models that rely on predefined feature extraction, our approach uses self-attention mechanisms to dynamically capture complex dependencies within and between flow-volume loops and volume-time curves. This enables the model to incorporate subtle pulmonary function characteristics that may precede the onset of clinical exacerbations, thereby enhancing predictive performance beyond conventional approaches.

We further analyzed the external validation cohort by dividing it into subgroups based on age. Approximately 42% of young patients with COPD experience moderate or severe exacerbations and about 16% experience severe exacerbations within the first 5 years [6]. However, most AE-COPD prediction models primarily target older COPD populations aged 40 and above, highlighting the need to validate prediction models in younger COPD populations [8]. The AI-PFT-Clin model demonstrated robust predictive performance across age subgroups, exhibiting particularly high performance in patients younger than 50 years. This may be because younger patients with COPD typically have fewer confounding factors, such as pulmonary or nonpulmonary comorbidities, that contribute to AE-COPD [25]. Moreover, young patients with COPD are less likely to have cognitive impairments or physical limitations, which can interfere with the accurate performance of spirometry, thereby increasing the reliability of lung function assessments [26]. Consequently, prediction models using spirometric information may have greater utility in younger patients.

The AI-PFT-Clin model outperformed the Clin model in predicting AE-COPD, even in patients without previous exacerbations. This improvement can be attributed primarily to the model’s enhanced sensitivity, allowing it to detect subtle physiological changes that may precede clinical exacerbations. By incorporating detailed features from flow-volume and volume-time curves of spirometry tests, the AI-PFT-Clin model captures small airway function and lung mechanics more comprehensively. These spirometric features may offer additional predictive power by identifying early physiological changes, such as small airway obstruction or reduced lung compliance, which are not yet apparent as clinical symptoms or through conventional spirometric parameters. The increased sensitivity of the AI-PFT-Clin model is particularly critical as it reduces the likelihood of false negatives, thereby identifying high-risk patients who may otherwise be overlooked by the Clin model. This is especially relevant in the context of COPD management, where early identification and intervention in high-risk individuals can prevent the progression to severe exacerbations, reduce health care usage, and ultimately improve long-term outcomes. By integrating both clinical data and comprehensive spirometric curve-derived features in an AI-driven framework, our study provides a more comprehensive risk assessment tool that may facilitate earlier and more precise clinical decision-making for patients with COPD.

This study has several limitations. First, the retrospective nature of the study may have introduced selection bias, as only patients with complete medical records and spirometry data were included, potentially limiting the generalizability of the findings. In addition, symptom data, including cough, dyspnea, and sputum production, were obtained retrospectively from medical records, which is a limitation as they were documented according to each physician’s individual clinical judgment rather than a standardized assessment framework. Second, the study population was derived from teaching hospitals, which may limit the generalizability of the findings to broader COPD populations. Third, while the AI-PFT-Clin model improved predictive performance, its accuracy for severe AE-COPD was relatively lower, likely due to its low incidence. Severe exacerbations also involve systemic factors beyond lung function, such as comorbidities and inflammation, which may not be fully captured by spirometry-based features alone. Fourth, in the Kaplan-Meier analysis, the proportion of patients remaining free from AE-COPD appeared to stabilize in later years. This is likely due to a combination of two factors, such as (1) high-risk patients experiencing earlier exacerbations, leaving behind a cohort with inherently lower risk; and (2) increasing censoring over time, leading to a reduction in the number of patients contributing to the estimates in later years. While this is a common characteristic of long-term survival analyses, caution is needed when interpreting findings beyond the mid-term follow-up due to the decreasing sample size over time. Fifth, the study did not account for dynamic changes in clinical variables and spirometry over time, which could provide additional insights into exacerbation risk.

### Conclusions

This study developed and validated an AI model integrating clinical data with spirometric information, including flow-volume loop and volume-time curve, to predict the risk of AE-COPD within 1 year. The AI-PFT-Clin model showed superior predictive performance compared to the Clin model, even in young patients with COPD and those without previous exacerbations. Further studies are needed to explore the potential of AI-based approaches using comprehensive spirometric data to enhance risk stratification and support more personalized management strategies in patients with COPD.

## Appendix

Multimedia Appendix 1 Data handling and model development.

Multimedia Appendix 2 Additional tables and figures.

## Abbreviations

AE: acute exacerbations
AE-COPD: acute exacerbations of chronic obstructive pulmonary disease
aHR: adjusted hazard ratio
AI: artificial intelligence
AUROC: area under the receiver operating characteristic curve
BRMH: Boramae Medical Center
CNN: convolutional neural network
COPD: chronic obstructive pulmonary disease
FEV1: forced expiratory volume in 1 second
FVC: forced vital capacity
ICS: inhaled corticosteroids
LABA: long-acting beta-agonists
LAMA: long-acting muscarinic antagonists
PFT: pulmonary function test
SNUH: Seoul National University Hospital
STROBE: Strengthening the Reporting of Observational Studies in Epidemiology

## Data Availability

The datasets generated or analyzed during this study are not publicly available due to concerns regarding the protection of patient privacy but are available from the corresponding author on reasonable request.
