# Supplementary material for: Deep Learning–Based Chronic Obstructive Pulmonary Disease Exacerbation Prediction Using Flow-Volume and Volume-Time Curve Imaging: Retrospective Cohort Study
Source: J Med Internet Res. 2025 May 15;27:e69785. doi: 10.2196/69785 (PMC12123229; doi:10.2196/69785)
Supplement: Multimedia Appendix 1 [file jmir_v27i1e69785_app1.docx]

**Supplementary information 1.** Data handling and model development

*Variables and measurements*

Demographic and clinical information were collected as baseline variables for analysis. These included age, gender, body mass index (BMI), smoking history (current smoking status and pack-years for ever-smokers), and symptoms such as cough, sputum, and dyspnea. Symptom information, including cough, dyspnea, and sputum production, was extracted from pulmonologist-documented medical records at the time of each spirometry assessment. Additionally, a history of moderate-to-severe AE-COPD and medical comorbidities were obtained. To account for the overall comorbidity burden, we used the Charlson Comorbidity Index rather than analyzing individual comorbid conditions separately. Laboratory data included measurements of white blood cell count (WBC), neutrophil and eosinophil counts and percentages, lymphocyte count and percentage, blood urea nitrogen (BUN), creatinine, total bilirubin, total protein, and albumin levels. Radiologic findings, such as emphysema and tuberculosis-destroyed lung, were identified based on radiologist reports.

Pulmonary function profiles were measured, including pre-bronchodilator (Pre-BDR) and post-bronchodilator (Post-BDR) FEV_1_, FVC, FEV_1_/FVC ratio, and forced expiratory flow at 25-75% (FEF_25-75%_). The diffusing capacity for carbon monoxide (DL_CO_) and DL_CO_ adjusted for alveolar volume (DL_CO_/VA) were also evaluated. Inhaled therapies were recorded, including the use of inhaled corticosteroids (ICS), long-acting beta-agonists (LABA), and long-acting muscarinic antagonists (LAMA).

*Extraction and Digitization of Flow-Volume Loops and Volume-Time Curves*

In the initial phase of image preprocessing, color channel decomposition was employed to separate the RGB images into their respective channels. Specifically, the blue and red channels, corresponding to the Pre-BDR and Post-BDR curves, respectively, were isolated from the flow-volume loop and volume-time curve images. Additionally, for flow-volume loops, inspiratory and expiratory components were distinguished based on their relative positions above and below the x-axis. Following channel separation, image segmentation was performed using thresholding techniques to isolate the relevant signals—namely, the flow-volume loops and volume-time curves—from the background. This involved setting appropriate pixel intensity thresholds to differentiate between the curve data and extraneous noise or artifacts present in the image.

Accurate extraction of the flow-volume loops and volume-time curves was achieved by determining the upper and lower boundaries of the curves through pixel intensity profile analysis across the columns of the segmented image. Specifically, the highest and lowest non-zero pixel values in each column were identified, representing the boundaries of the curves. To reduce the influence of noise and irregularities in the raw pixel data, Gaussian filtering was applied, providing a smooth, continuous representation of the curves by attenuating high-frequency noise. During the curve extraction process, cubic spline interpolation was utilized to reconstruct any missing or discontinuous segments in the curves, ensuring smooth transitions between data points while maintaining the overall geometric integrity of the curve. Furthermore, any anomalies detected, such as abrupt shifts or discontinuities, were addressed using adaptive thresholding and localized corrections.

Subsequent to extraction, the discrete pixel data representing the flow-volume loops and volume-time curves were converted into continuous data points using linear interpolation. This ensured a coherent and accurate representation of the underlying physiological data by producing continuous curves from the extracted discrete points. Finally, the extracted and smoothed curves were aligned based on reference points, specifically the starting points of each curve, and scaled according to the axis values derived from the image. This process ensured that the final digitized curves accurately represented the physiological parameters of interest.

*AI-PFT Model Development*

The AI-PFT model is designed to process two primary inputs: volume-time curves and flow-volume loops. In the flow-volume loop, both the inspiratory and expiratory phases of the pre-BDR and post-BDR curves were analyzed, while only the inspiratory phases were included for the volume-time curve analysis. The flow-volume loops and volume-time curves were normalized to each patient's predicted value using the reference curves, which were provided by spirometric equipment (Vmax 22 system, CareFusion, San Diego, California).

Each of these inputs is structured as a sequence of measurements, with shapes of (100, 2) for the volume-time curves and (120, 4) for the flow-volume loops, respectively. Volume-time curves are embedded using a tubelet embedding layer that segments the input into patches of size (20×2). This layer extracts local spatial features, creating a patch-wise representation of the curve data. Flow-volume loops, on the other hand, are divided into patches of size (12×4), using a similar tubelet embedding layer. The patch sizes were determined using the grid search method to minimize the mean squared error on the validation set. This process utilized masked image modeling, a self-supervised learning approach.(Chen et al., 2023) The main model was constructed as an autoencoder architecture with a transformer employed as the encoder and a multi-layer perceptron (MLP) with a single dense layer serving as the decoder. The embedding process is crucial for capturing local dependencies within the time-series data, allowing the subsequent Transformer to better model both short-term and long-term relationships in the input sequences. After the initial embedding, each patch undergoes a patch encoding process. This involves the addition of positional encodings to preserve the order of the patches, which is particularly important for sequential data like respiratory curves. The positional encoding helps the model understand the sequence of the patches within each curve. Both the volume-time curves and flow-volume loops are passed through separate 1D Transformer layers. Each Transformer consists of stacked Multi-Head Self-Attention layers, implemented using Scaled Dot-Product Attention to capture the dependencies between different patches.(Vaswani, 2017) The Transformers output a refined feature representation for each type of curve by leveraging both local and global contextual information.

A Scaled Dot-Product Self-Attention mechanism is applied to the features extracted from both the volume-time and flow-volume loops. This step combines the information from the two distinct types of curves, allowing the model to capture interactions between the volume-time and flow-volume representations. By scaling the dot products, the model prevents gradient vanishing/explosion and enhances its capacity to model the relationships within the data. The resulting feature maps from each curve type are then passed through Global Average Pooling 1D layers, which serve to reduce the dimensionality of the data while retaining its global contextual features. This pooling operation outputs a compact vector representation of the extracted features. After pooling, the feature representations from both input streams (volume-time curves and flow-volume loops) are combined using an add operation. This fused representation is then passed through a layer normalization layer to ensure that the combined features are well-scaled and have normalized statistics, thus preventing internal covariate shifts during the training process.

Following normalization, the fused feature vector is passed through two separate Dense layers, each corresponding to one of the target outcomes. These Dense layers form the core of the model’s multi-layer perceptrons (MLPs). One MLP head is responsible for predicting the probability of moderate to severe AE. The other MLP head focuses on predicting the probability of severe AE. Both MLPs consist of fully connected layers interspersed with dropout layers to prevent overfitting, especially given the high-dimensional nature of the input data. The output from each MLP is a probability score, generated through an output layer using a sigmoid activation function. This results in two predicted probabilities: one for moderate to severe AE and another for severe AE.

The guiding metric in model training was the area under the receiver operating characteristic curve. The focal cross-entropy loss function was employed, along with the AdamW optimizer. A dropout rate of 0.5 was applied to all dropout layers within the MLP, while a rate of 0.2 was used for other layers. An early stopping mechanism based on the monitoring of validation loss was implemented to prevent overfitting. Any unspecified hyperparameters were set to their default values. Furthermore, learning rate scheduling was utilized to adjust the optimizer dynamically, ensuring stable convergence throughout the training process.

## References

Chen Z, Agarwal D, Aggarwal K, et al. Masked image modeling advances 3d medical image analysis. Proceedings of the IEEE/CVF Winter Conference on Applications of Computer Vision 2023.

Vaswani, A. Attention is all you need. Advances in neural information processing systems 2017
