# Supplementary material for: Deep Learning–Based Chronic Obstructive Pulmonary Disease Exacerbation Prediction Using Flow-Volume and Volume-Time Curve Imaging: Retrospective Cohort Study
Source: J Med Internet Res. 2025 May 15;27:e69785. doi: 10.2196/69785 (PMC12123229; doi:10.2196/69785)
Supplement: Multimedia Appendix 2 [file jmir_v27i1e69785_app2.docx]

**Supplementary materials**

**Figure S1.** The architecture of the AI-PFT model

**
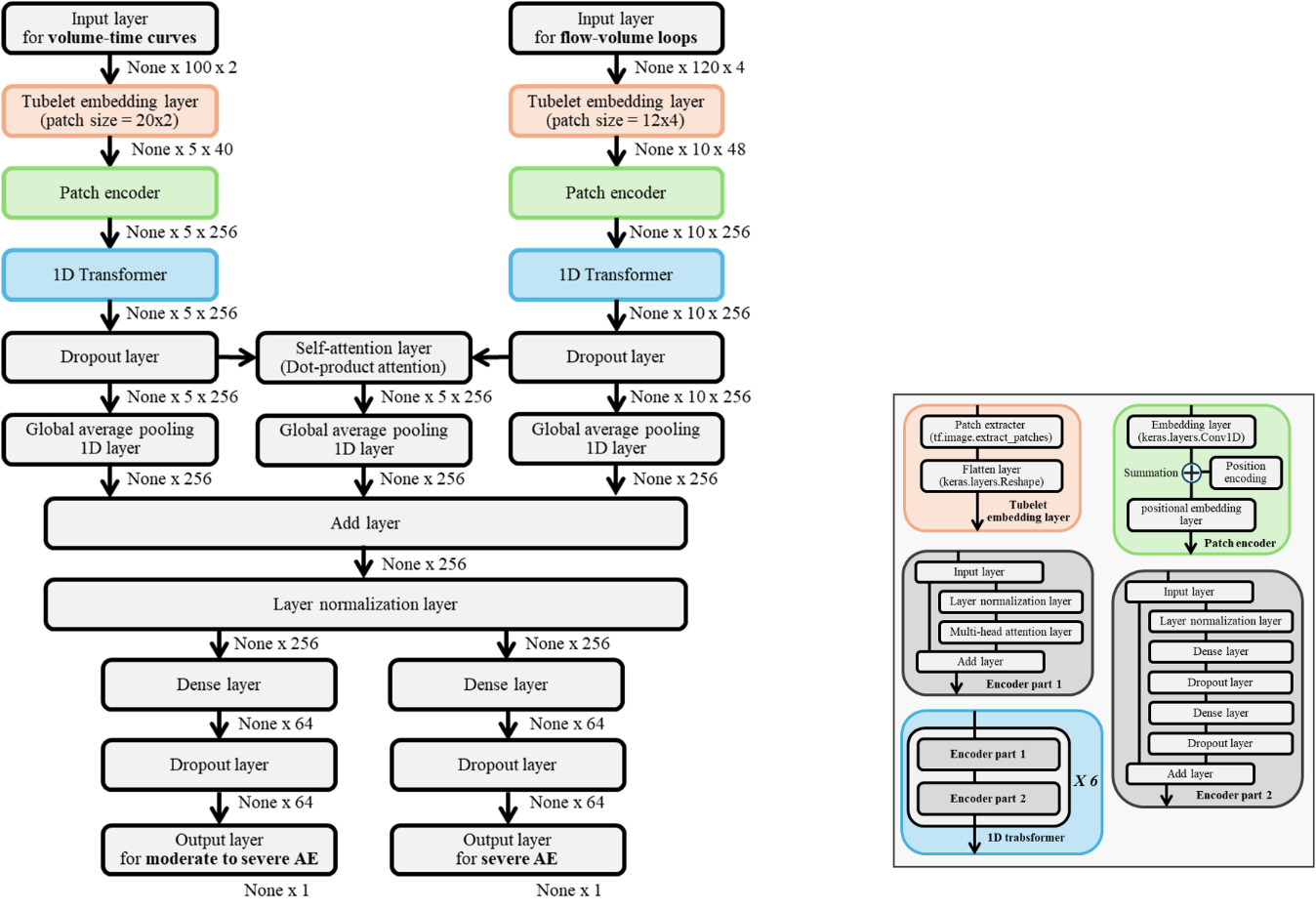
**

Abbreviations: AE, acute exacerbation.

| **Table S1.** Baseline characteristics of internal test set, young, and old patient groups | | | | | |
| --- | --- | --- | --- | --- | --- |
|  | **Variable** | **BRMH Internal test set (n = 204)** | **< 50 years old at SNUH (n = 149)** | **≥ 50 years old at SNUH (n = 340)** | ***P* value** |
| **Demographics** | | | | | |
|  | Age, yrs, median [IQR] | 64 [52 – 72] | 43 [37 – 46] | 63 [56 – 68] | < 0.001*** |
|  | Male, n (%) | 171 (83.8 %) | 93 (62.4 %) | 243 (71.5 %) | < 0.001*** |
|  | BMI, kg/m^2^, median [IQR] | 22.4 [19.9 – 24.4] | 22.5 [20.4 – 25.5] | 22.4 [22.0 – 23.0] | 0.568 |
|  | Smoking, n (%) |  |  |  | < 0.001*** |
|  | Never smoker | 49 (24.0 %) | 69 (46.3 %) | 67 (19.7 %) |  |
|  | Ex-smoker | 88 (43.1 %) | 66 (44.3 %) | 252 (74.1 %) |  |
|  | Current smoker | 67 (32.8 %) | 14 (9.4 %) | 21 (6.2 %) |  |
|  | Pack-year in ever-smokers, median [IQR] | 30.0 [5.0 – 45.0] | 15.3 [0.5 – 22.2] | 33.3 [25.0 – 37.2] | < 0.001*** |
| **Respiratory symptoms** | |  |  |  |  |
|  | Cough, n (%) | 86 (42.2 %) | 8 (5.4 %) | 48 (14.1 %) | < 0.001*** |
|  | Sputum, n (%) | 106 (52.0 %) | 42 (28.2 %) | 55 (16.2 %) | < 0.001*** |
|  | Dyspnea, n (%) | 172 (84.3 %) | 78 (52.3 %) | 317 (93.2 %) | < 0.001*** |
| **History of moderate-to-severe AE-COPD , n (%)** | | 96 (47.1 %) | 42 (28.2 %) | 258 (75.9 %) | < 0.001*** |
| **Charlson comorbidity index, median [IQR]** | | 2 [1 – 3] | 1 [1 – 1] | 2 [1 – 2] | < 0.001*** |
| **Laboratory findings** | | | | | |
|  | Blood WBC, 10^3^/μL, median [IQR] | 7316 [6116 – 8972] | 7240 [5848 – 8408] | 7692 [7587 – 7753] | 0.040* |
|  | Blood neutrophil, %, median [IQR] | 59.6 [55.0 – 67.8] | 57.4 [51.8 – 62.2] | 60.7 [60.2 – 61.3] | < 0.001*** |
|  | Blood neutrophil count, 10^3^/μL, median [IQR] | 4538 [3474 – 5688] | 4112 [3258 – 5024] | 4820 [4775 – 4852] | < 0.001*** |
|  | Blood lympho, %, median [IQR] | 29.4 [22.5 – 34.9] | 30.9 [26.6 – 35.1] | 28.8 [28.4 – 29.2] | < 0.001*** |
|  | Blood lympho count, 10^3^/μL, median [IQR] | 2052 [1654 – 2578] | 2264 [1707 – 2590] | 2093 [2040 – 2134] | 0.016* |
|  | Blood eosinophil, %, median [IQR] | 2.70 [1.18 – 3.93] | 3.40 [1.70 – 5.20] | 2.96 [2.70 – 3.17] | 0.002** |
|  | Blood eosinophil count, 10^3^/μL, median [IQR] | 188 [88 – 289] | 229 [118 – 348] | 213 [188 – 231] | 0.025* |
|  | BUN, mg/dL, median [IQR] | 14.6 [12.0 – 17.0] | 12.0 [10.0 – 14.0] | 14.9 [13.8 – 15.6] | < 0.001*** |
|  | Creatinine, mg/dL, median [IQR] | 0.89 [0.79 – 0.99] | 0.90 [0.73 – 1.00] | 0.92 [0.90 – 0.94] | 0.009** |
|  | Total bilirubin, mg/dL, median [IQR] | 0.74 [0.60 – 0.90] | 0.70 [0.50 – 0.90] | 0.75 [0.74 – 0.75] | 0.132 |
|  | Blood protein, g/dL, median [IQR] | 7.00 [6.60 – 7.30] | 7.30 [7.02 – 7.80] | 7.03 [6.96 – 7.12] | < 0.001*** |
|  | Blood albumin, g/dL, median [IQR] | 4.10 [3.87 – 4.30] | 4.30 [4.10 – 4.50] | 4.06 [4.02 – 4.12] | < 0.001*** |
| **Radiologic findings** | | | | | |
|  | Emphysema, n (%) | 115 (56.4 %) | 30 (20.1 %) | 43 (12.6 %) | < 0.001*** |
|  | TDL, n (%) | 24 (11.8 %) | 12 (8.1 %) | 8 (2.4 %) | < 0.001*** |
| **Pulmonary function tests** | | | | | |
|  | Post-BDR FEV_1_, L, median [IQR] | 1.79 [1.32 – 2.19] | 2.09 [1.58 – 2.66] | 1.65 [1.41 – 1.81] | < 0.001*** |
|  | Post-BDR FEV_1_, %, median [IQR] | 70.0 [56.0 – 81.2] | 72.0 [51.0 – 81.0] | 66.1 [60.8 – 67.4] | < 0.001*** |
|  | Post-BDR FVC, L, median [IQR] | 3.23 [2.55 – 3.84] | 3.57 [2.88 – 4.40] | 3.16 [2.85 – 3.39] | < 0.001*** |
|  | Post-BDR FVC, %, median [IQR] | 87.0 [76.0 – 98.0] | 72.0 [51.0 – 83.0] | 85.1 [80.2 – 87.6] | < 0.001*** |
|  | Post-BDR FEV_1_/FVC, %, median [IQR] | 59.0 [48.0 – 67.0] | 61.0 [55.1 – 66.0] | 54.8 [53.1 – 56.6] | < 0.001*** |
|  | Post-BDR FEF_25-75%_, L/sec, median [IQR] | 0.79 [0.49 – 1.21] | 1.19 [0.95 – 1.46] | 0.81 [0.68 – 0.90] | < 0.001*** |
|  | Post-BDR FEF_25-75%_, %, median [IQR] | 30.0 [20.0 – 44.0] | 37.5 [28.6 – 45.3] | 30.7 [28.5 – 32.1] | < 0.001*** |
|  | DL_CO_, %, median [IQR] | 86.9 [70.0 – 97.0] | 86.0 [72.8 – 98.4] | 81.9 [76.4 – 87.2] | < 0.001*** |
|  | DL_CO_/VA, %, median [IQR] | 88.0 [71.8 – 108.2] | 111.1 [102.2 – 121.9] | 88.3 [79.5 – 101.0] | < 0.001*** |
| **Inhaled treatments** | | | | | |
|  | LABA, n (%) | 142 (69.6 %) | 28 (18.8 %) | 329 (96.8 %) | < 0.001*** |
|  | LAMA, n (%) | 154 (75.5 %) | 67 (45.0 %) | 331 (97.4 %) | < 0.001*** |
|  | ICS, n (%) | 113 (55.4 %) | 78 (52.3 %) | 290 (85.3 %) | < 0.001*** |
| Abbreviations: AE, acute exacerbation; BUN, blood urea nitrogen; DLCO, Diffusing capacity of the Lung for CO; FEF, forced expiratory flow; FEV, forced expiratory volume; FEV1, Forced Expiratory Volume in one second; FVC, Forced Vital Capacity; Hx., history; ICS, inhaled corticosteroid; LABA, long-acting beta-agonist; LAMA, long-acting muscarinic antagonist; Post-BDR, post-bronchodilator Response; TDL, tuberculosis destroyed lung; VA, alveolar volume; WBC, white blood cell. | | | | | |

| **Table S2.** Detailed information on the performance of predictive models | | | | | | | |
| --- | --- | --- | --- | --- | --- | --- | --- |
|  | Cohort | Models | AUROC [95% CI] | Sensitivity | Specificity | PPV | NPV |
| **Prediction of moderate-to-severe exacerbations** | | | |  |  |  |  |
|  | Internal test set | Clin model | 0.719 [0.686 – 0.752] | 0.441 [0.383 – 0.499] | 0.897 [0.881 – 0.913] | 0.530 [0.470 – 0.591] | 0.858 [0.837 – 0.880] |
|  |  | AI-PFT model | 0.664 [0.631 – 0.698] | 0.391 [0.340 – 0.442] | 0.790 [0.767 – 0.813] | 0.330 [0.284 – 0.376] | 0.831 [0.809 – 0.852] |
|  |  | AI-PFT-Clin model | 0.745 [0.712 – 0.778] | 0.600 [0.543 – 0.658] | 0.778 [0.755 – 0.801] | 0.417 [0.372 – 0.462] | 0.880 [0.859 – 0.902] |
|  | External validation set | Clin model | 0.730 [0.713 – 0.746] | 0.505 [0.476 – 0.533] | 0.850 [0.837 – 0.863] | 0.576 [0.544 – 0.609] | 0.809 [0.795 – 0.824] |
|  |  | AI-PFT model | 0.635 [0.615 – 0.654] | 0.656 [0.628 – 0.685] | 0.548 [0.530 – 0.566] | 0.370 [0.350 – 0.390] | 0.798 [0.779 – 0.817] |
|  |  | AI-PFT-Clin model | 0.755 [0.738 – 0.772] | 0.763 [0.737 – 0.789] | 0.588 [0.571 – 0.606] | 0.428 [0.409 – 0.448] | 0.860 [0.844 – 0.876] |
| **Prediction of severe exacerbations** | | |  |  |  |  |  |
|  | Internal test set | Clin model | 0.742 [0.691 – 0.793] | 0.496 [0.397 – 0.596] | 0.894 [0.878 – 0.910] | 0.273 [0.210 – 0.336] | 0.957 [0.946 – 0.967] |
|  |  | AI-PFT model | 0.796 [0.756 – 0.835] | 0.727 [0.642 – 0.811] | 0.697 [0.675 – 0.719] | 0.161 [0.130 – 0.192] | 0.970 [0.959 – 0.980] |
|  |  | AI-PFT-Clin model | 0.791 [0.742 – 0.839] | 0.733 [0.649 – 0.816] | 0.694 [0.672 – 0.716] | 0.161 [0.129 – 0.193] | 0.970 [0.960 – 0.980] |
|  | External validation set | Clin model | 0.675 [0.647 – 0.703] | 0.453 [0.399 – 0.506] | 0.788 [0.773 – 0.802] | 0.158 [0.131 – 0.186] | 0.942 [0.934 – 0.950] |
|  |  | AI-PFT model | 0.673 [0.641 – 0.704] | 0.832 [0.791 – 0.873] | 0.378 [0.363 – 0.394] | 0.106 [0.091 – 0.120] | 0.962 [0.953 – 0.971] |
|  |  | AI-PFT-Clin model | 0.713 [0.683 – 0.744] | 0.838 [0.796 – 0.880] | 0.428 [0.409 – 0.448] | 0.115 [0.098 – 0.131] | 0.968 [0.959 – 0.976] |

The values are expressed as mean [95% CI].
Abbreviations: AUROC, area under the receiver operating characteristic curve; NPV, negative predictive value; PPV, positive predictive value.

**Figure S2.** Distribution of AI-PFT-Clin score in internal test and external validation sets

**
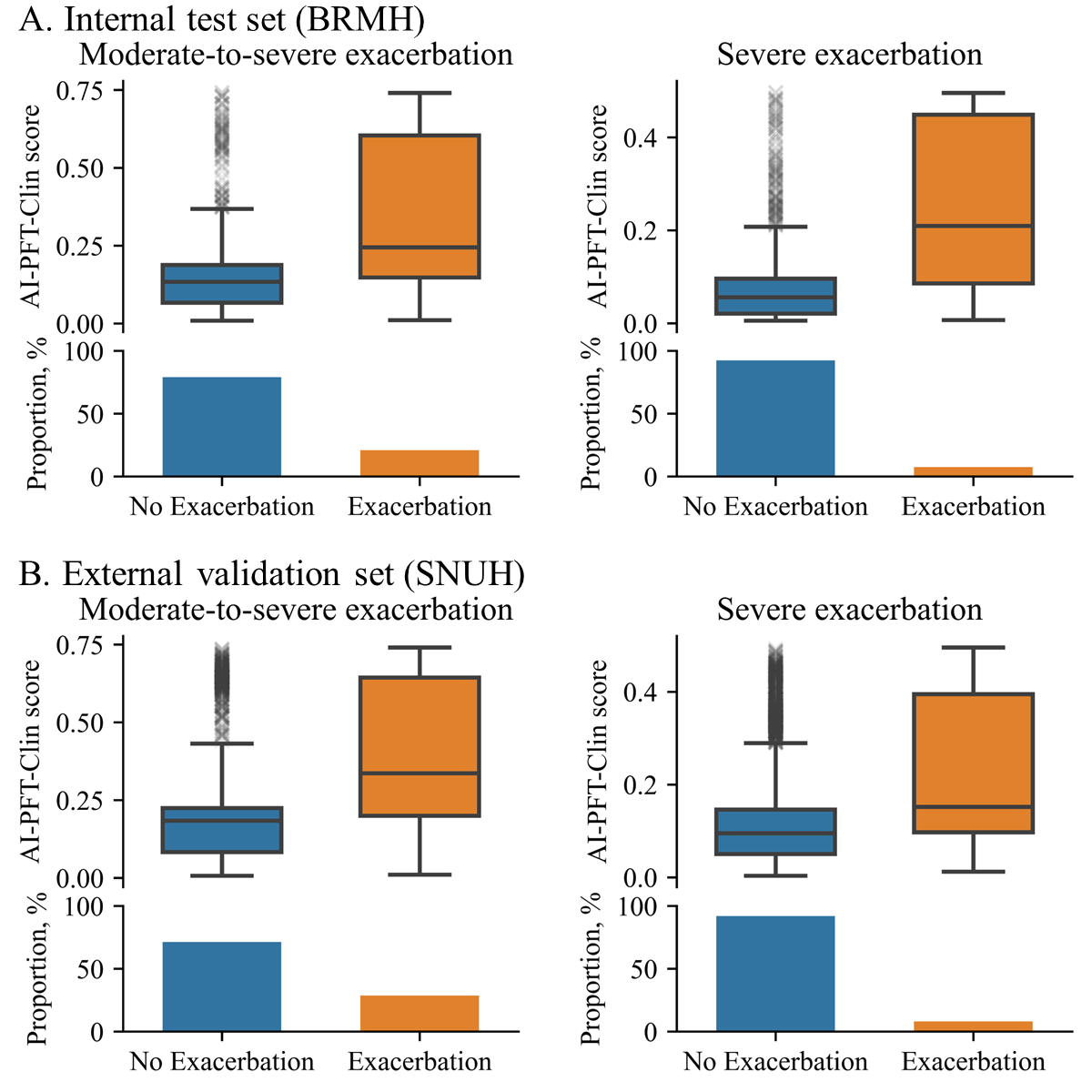
**

The whiskers of the box plots are 1.5 times the interquartile ranges.

**Figure S3.** Subgroup analyses for predicting moderate-to-severe exacerbations


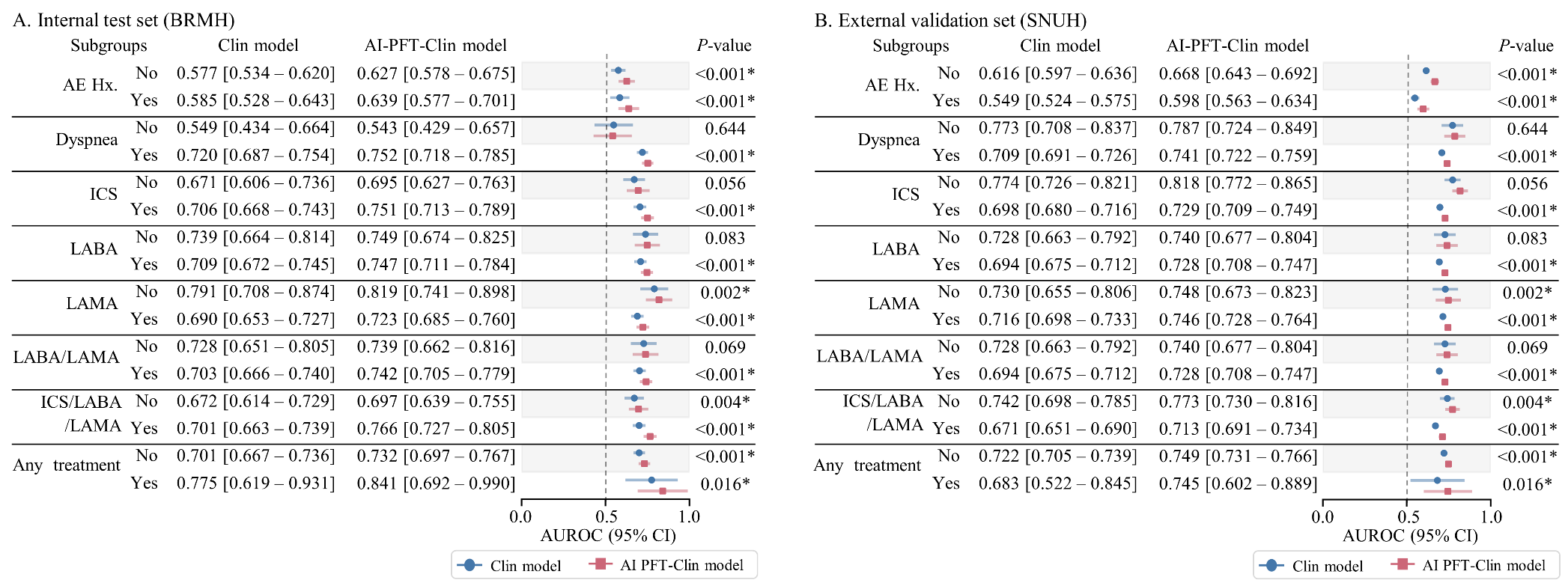


Abbreviations: AE, acute exacerbation; ICS, inhaled corticosteroid; LABA, long-acting beta-agonist; LAMA, long-acting muscarinic antagonist.

**Figure S4.** Subgroup analyses for predicting severe exacerbations


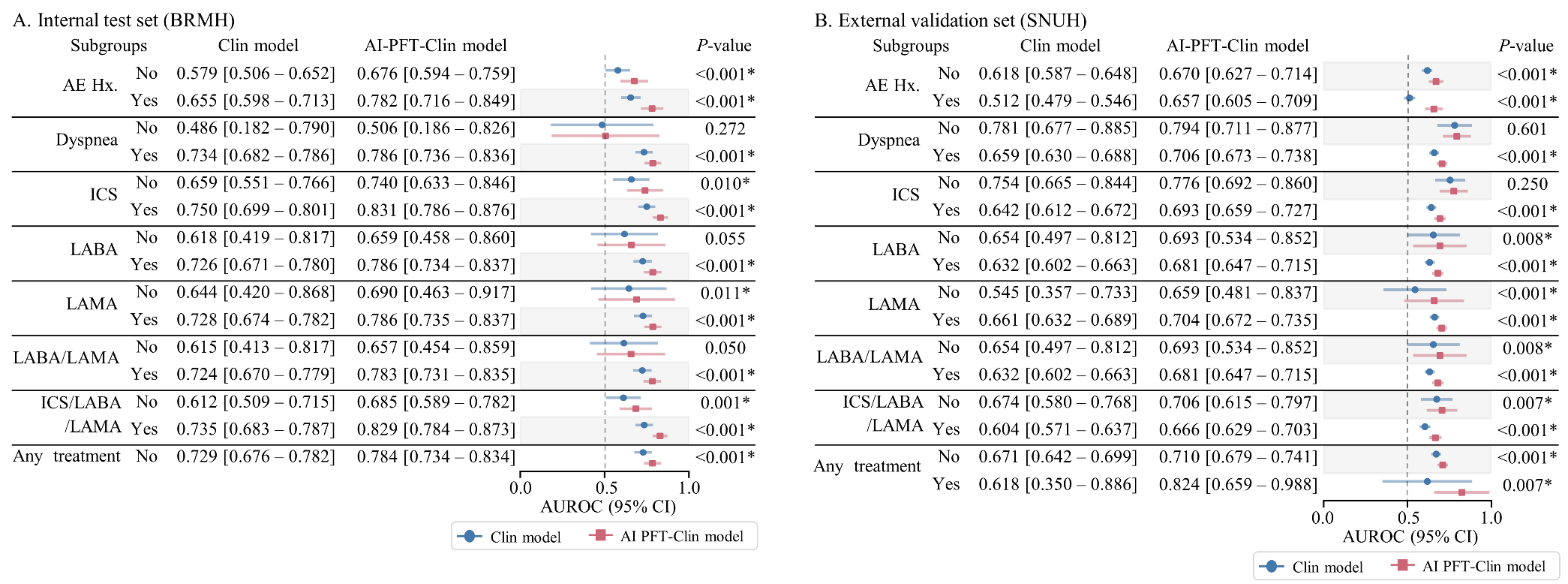


Abbreviations: AE, acute exacerbation; ICS, inhaled corticosteroid; LABA, long-acting beta-agonist; LAMA, long-acting muscarinic antagonist.

| **Table S3.** Detailed information on the performance of predictive models according to age | | | | | | | |
| --- | --- | --- | --- | --- | --- | --- | --- |
|  | Cohort | Models | AUROC [95% CI] | Sensitivity | Specificity | PPV | NPV |
| **Prediction of moderate-to-severe exacerbations** | | | |  |  |  |  |
|  | < 50  yeras old | Clin model | 0.710 [0.652 – 0.768] | 0.164 [0.091 – 0.236] | 0.951 [0.936 – 0.967] | 0.266 [0.143 – 0.390] | 0.914 [0.893 – 0.935] |
|  |  | AI-PFT model | 0.640 [0.573 – 0.707] | 0.503 [0.408 – 0.599] | 0.723 [0.690 – 0.755] | 0.163 [0.115 – 0.212] | 0.931 [0.912 – 0.951] |
|  |  | AI-PFT-Clin model | 0.732 [0.674 – 0.790] | 0.254 [0.169 – 0.339] | 0.926 [0.909 – 0.944] | 0.270 [0.177 – 0.364] | 0.920 [0.900 – 0.941] |
|  | ≥ 50  yeras old | Clin model | 0.686 [0.667 – 0.705] | 0.531 [0.500 – 0.562] | 0.813 [0.797 – 0.829] | 0.594 [0.563 – 0.626] | 0.771 [0.755 – 0.788] |
|  |  | AI-PFT model | 0.603 [0.581 – 0.625] | 0.667 [0.637 – 0.696] | 0.481 [0.457 – 0.505] | 0.398 [0.374 – 0.421] | 0.737 [0.713 – 0.762] |
|  |  | AI-PFT-Clin model | 0.719 [0.699 – 0.739] | 0.805 [0.777 – 0.832] | 0.458 [0.435 – 0.482] | 0.433 [0.412 – 0.455] | 0.820 [0.795 – 0.846] |
| **Prediction of severe exacerbations** | | |  |  |  |  |  |
|  | < 50  yeras old | Clin model | 0.814 [0.683 – 0.945] | 0.331 [0.018 – 0.644] | 0.956 [0.942 – 0.969] | 0.077 [-0.002 – 0.156] | 0.992 [0.986 – 0.998] |
|  |  | AI-PFT model | 0.793 [0.689 – 0.897] | 0.691 [0.392 – 0.990] | 0.634 [0.600 – 0.667] | 0.021 [0.005 – 0.037] | 0.995 [0.989 – 1.000] |
|  |  | AI-PFT-Clin model | 0.857 [0.745 – 0.970] | 0.587 [0.240 – 0.933] | 0.864 [0.843 – 0.885] | 0.046 [0.009 – 0.084] | 0.995 [0.989 – 1.000] |
|  | ≥ 50  yeras old | Clin model | 0.609 [0.577 – 0.641] | 0.455 [0.395 – 0.514] | 0.738 [0.721 – 0.756] | 0.163 [0.136 – 0.190] | 0.924 [0.912 – 0.935] |
|  |  | AI-PFT model | 0.641 [0.605 – 0.677] | 0.835 [0.793 – 0.877] | 0.300 [0.284 – 0.317] | 0.118 [0.103 – 0.133] | 0.942 [0.927 – 0.957] |
|  |  | AI-PFT-Clin model | 0.659 [0.623 – 0.694] | 0.845 [0.805 – 0.885] | 0.295 [0.278 – 0.312] | 0.118 [0.104 – 0.133] | 0.944 [0.929 – 0.959] |

The values are expressed as mean [95% CI].
Abbreviations: AUROC, area under the receiver operating characteristic curve; NPV, negative predictive value; PPV, positive predictive value.

**Table S4.** Subgroup analyses for predicting moderate-to-severe exacerbations according to age


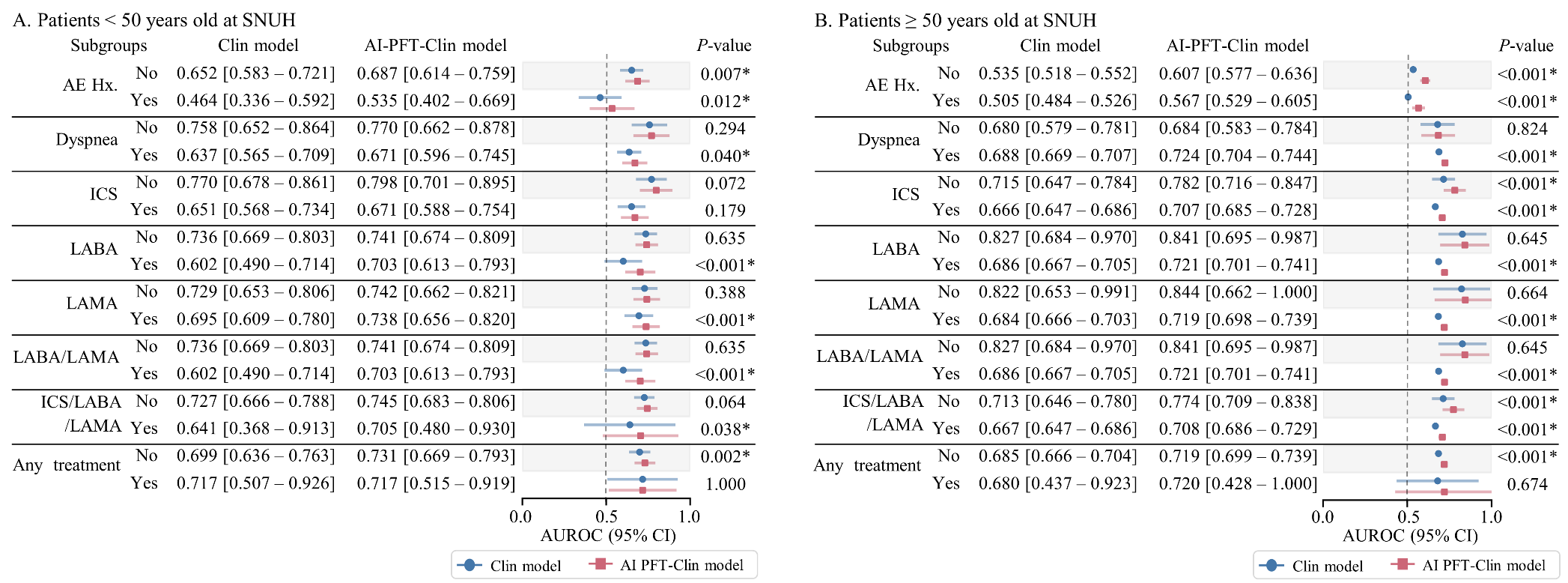


Abbreviations: AE, acute exacerbation; ICS, inhaled corticosteroid; LABA, long-acting beta-agonist; LAMA, long-acting muscarinic antagonist.

**Figure S5.** Subgroup analyses for predicting severe exacerbations according to age


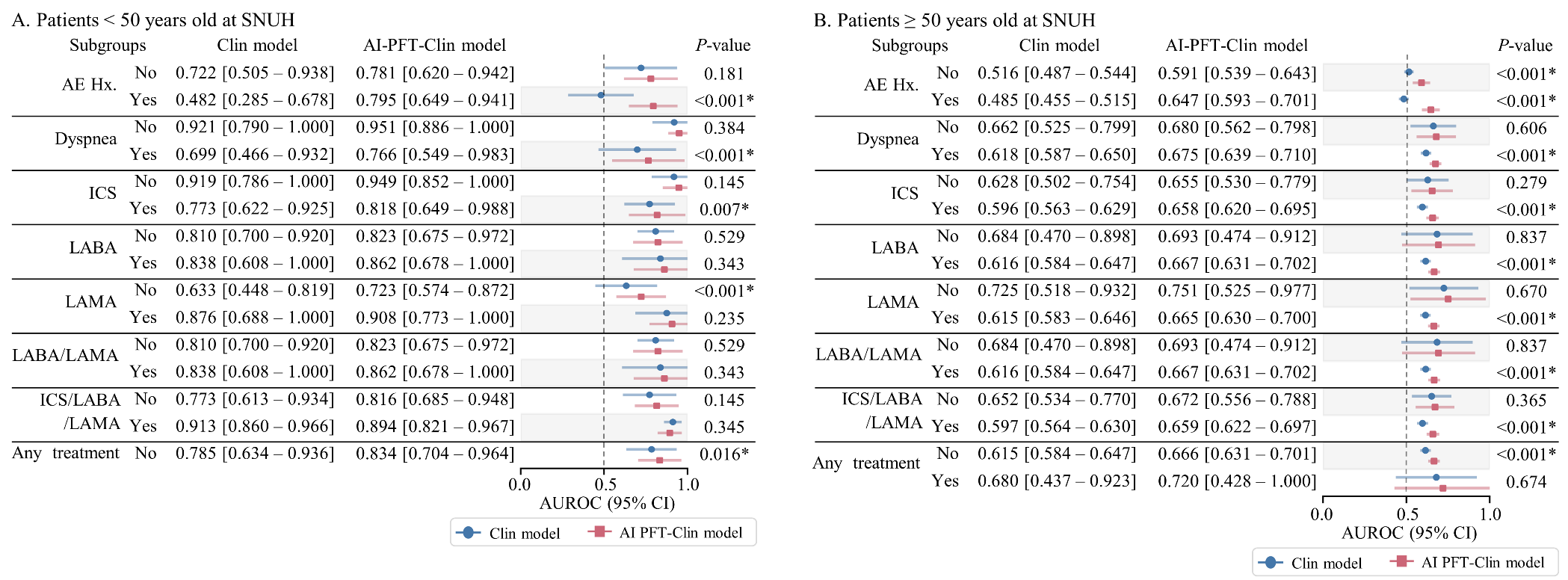


Abbreviations: AE, acute exacerbation; ICS, inhaled corticosteroid; LABA, long-acting beta-agonist; LAMA, long-acting muscarinic antagonist.

**Figure S6.** Time to exacerbation event according to AI-PFT-Clin score in different age groups


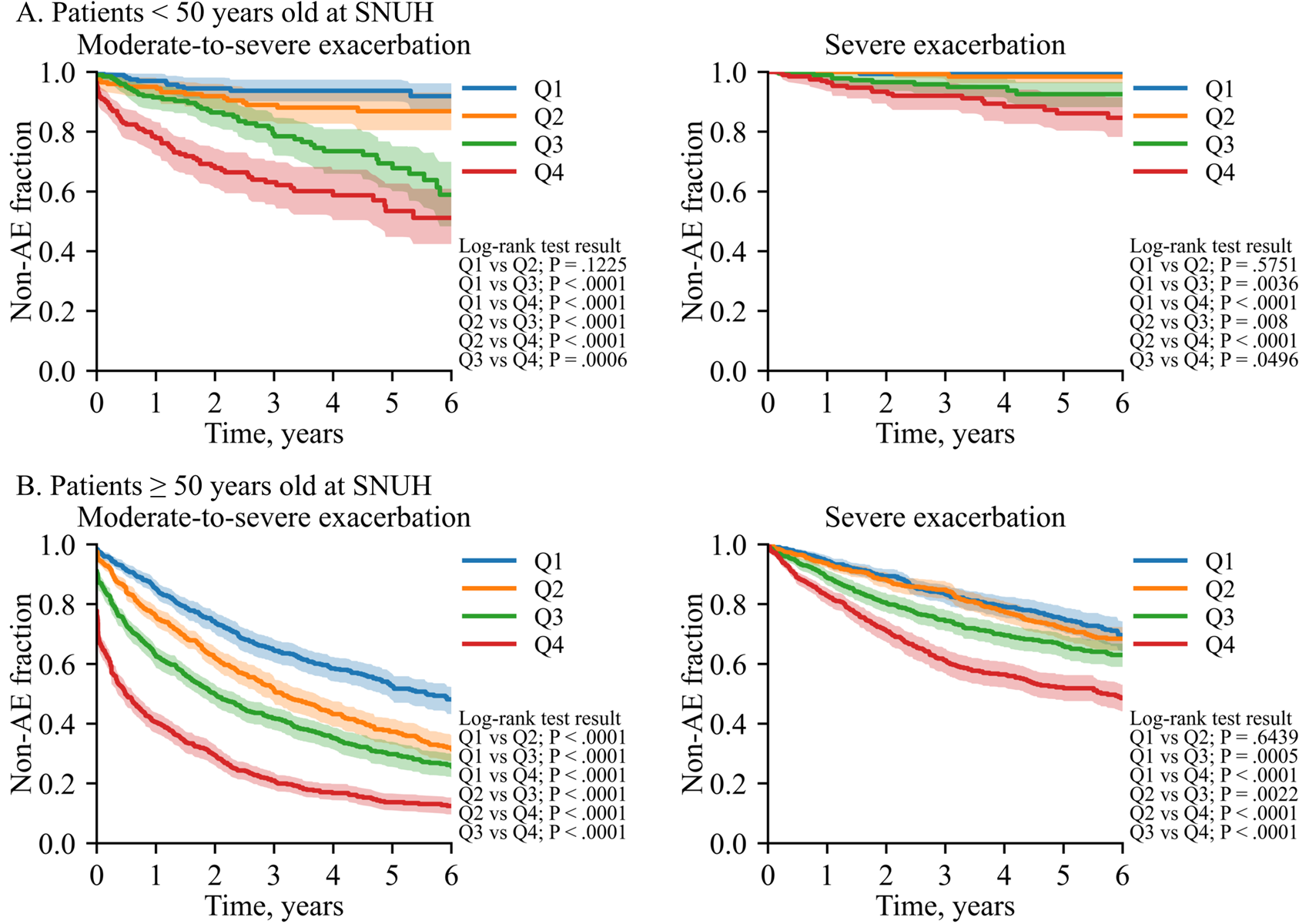


AI-PFT-Clin score was used to obtain the four strata. According to the prediction score, Q1, Q2, Q3, and Q4 denote the first, second, third, and fourth quartiles. Solid lines and shades represent mean curves and 95% confidence interval areas, respectively. Pair-wise comparisons between the curves were performed and *P* values are presented in the legend. Abbreviations: AE, acute exacerbation.

**Figure S7.** Predicted probability trajectories of exacerbation according to age over 6 years


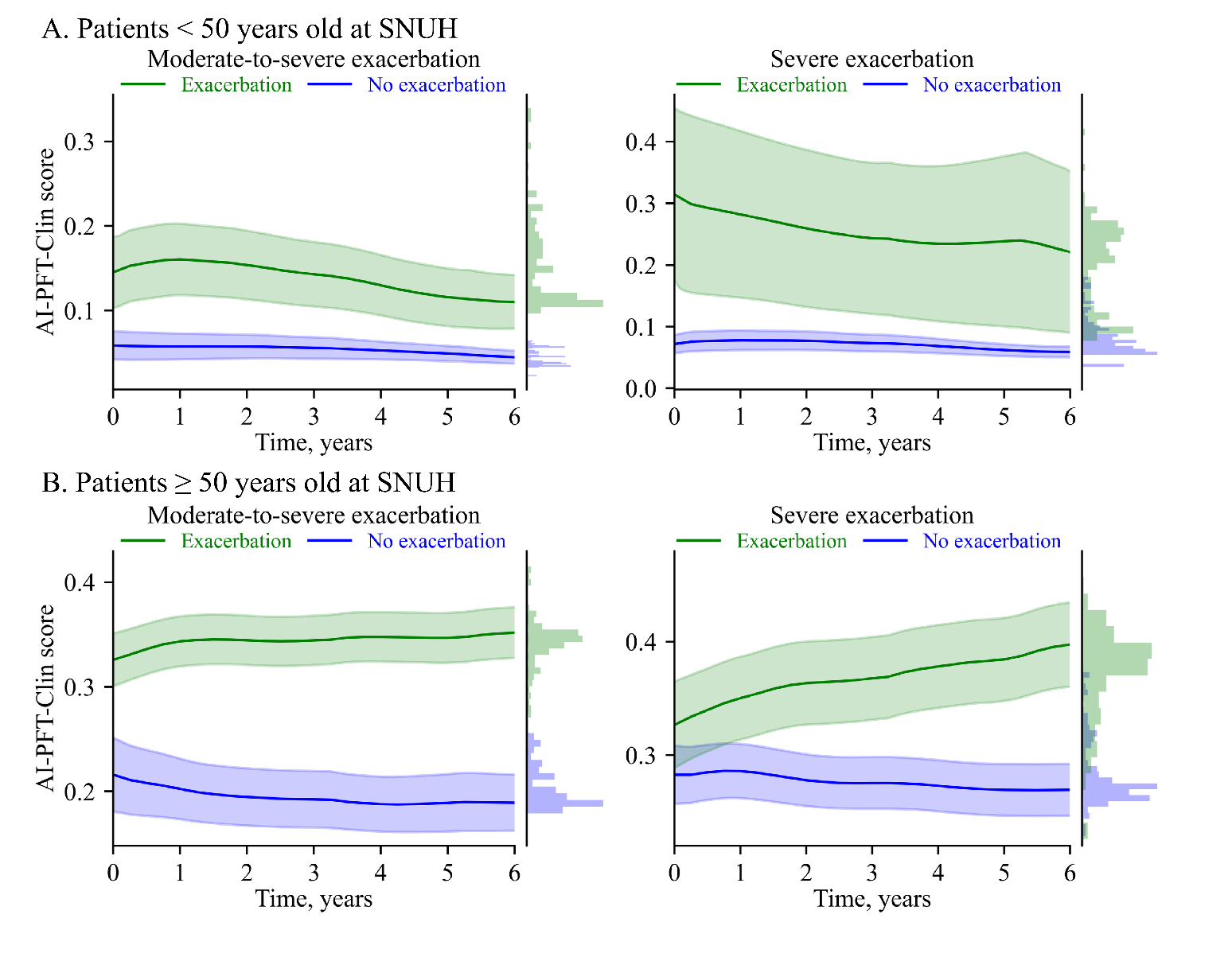


The predicted probabilities were obtained from the AI-PFT-Clin model. Solid lines and shades represent mean curves and 95% confidence interval areas, respectively. Histograms on the right axis indicate distributions of the predicted probability.
